# Down-regulation of microsomal prostaglandin E2 synthase-1 in the infrapatellar fat pad of osteoarthritis patients with hypercholesterolemia

**DOI:** 10.1186/s12944-018-0792-7

**Published:** 2018-06-13

**Authors:** Manabu Mukai, Kentaro Uchida, Shotaro Takano, Dai Iwase, Jun Aikawa, Gen Inoue, Masayuki Miyagi, Masashi Takaso

**Affiliations:** Department of Orthopedic Surgery, Kitasato University School of Medicine, 1-15-1 Minami-ku Kitasato, Sagamihara City, Kanagawa 252-0374 Japan

**Keywords:** Hypercholesterolemia, Infrapatellar fat pad, Microsomal prostaglandin E2 synthase-1

## Abstract

**Background:**

While epidemiological studies have reported a potential role for hypercholesterolemia (HCE) in osteoarthritis (OA), the association between HCE and OA has yet to be clarified. Adipose tissue is a primary locus for cholesterol metabolism and the presence of HCE reportedly causes adipose dysfunction. The knee joint contains adipose tissue in the form of the infrapatellar fat pad (IPFP), which has been shown to contribute to the pathophysiology of OA in the knee via the secretion of inflammatory mediators. However, the effect of HCE on the expression of inflammatory mediators in the IPFP has not been elucidated.

**Methods:**

IPFP and synovial tissues (ST) were extracted from 145 subjects with OA, diagnosed by radiography, during total knee arthroplasty. OA patients were divided into three groups according to their total cholesterol levels (Desirable, Borderline high and High) based on the National Cholesterol Education Program Adult Treatment Panel III (NCEPATP III). We examined the expression of cyclooxygenase-2 (COX-2), microsomal prostaglandin E synthase-1 (mPGES1), tumor necrosis factor (TNF)-α, interleukin (IL)-1β, and IL-6 using real-time polymerase chain reaction and compared results among the Desirable, Borderline high and High groups.

**Results:**

The mRNA expression levels of TNF-α, IL-1β, and IL-6 in ST and the IPFP were not significantly different among the three groups. COX-2 mRNA expression in ST and IPFP was likewise not different among the three groups. While the mRNA expression level of mPGES1 in ST was also not significantly different, that of mPGES1 in the IPFP was significantly lower in the High group than in the Desirable and Borderline high groups.

**Conclusion:**

mRNA levels of mPGES-1 are reduced in the IPFP of knee OA patients with HCE. Additional studies are need to clarify the effect of mPGES-1 down-regulation in OA pathology.

**Electronic supplementary material:**

The online version of this article (10.1186/s12944-018-0792-7) contains supplementary material, which is available to authorized users.

## Background

Numerous reports have suggested that osteoarthritis (OA), rather than simply being a mechanical stress-related joint disorder, is also a metabolic syndrome wherein various risk factors function together to cause disease initiation and/or development. While hypercholesterolemia (HCE) is an established risk factor for cardiovascular disorders, epidemiological studies have also reported a possible role for HCE in OA [1–4]. HCE is associated with both unilateral and bilateral knee OA independently of obesity [5]. High serum cholesterol levels are associated with both knee and generalized OA [4]. However, the association between HCE and OA has yet to be clarified.

Adipose tissue is a primary locus for cholesterol metabolism, and the presence of HCE reportedly causes adipose dysfunction. The knee joint contains adipose tissue in the form of the infrapatellar fat pad (IPFP), which is intracapsularly and extrasynovially positioned near the synovium, cartilage and bone [6]. Its location within the joint makes it possible for the IPFP to play a role in the pathophysiology of OA in the knee via the secretion of inflammatory signals such as interleukin (IL)-6 and tumor necrosis factor-a (TNF-α) [7, 8]. However, the effect of HCE on inflammatory cytokine expression in the IPFP has not been elucidated.

Prostaglandin E2 (PGE2) is synthesized by the arachidonic acid cascade, which is formed by the concerted action of cyclooxygenases-2 (COX-2) and the specific terminal synthase microsomal prostaglandin E2 synthase-1 (mPGES-1) [9, 10]. PGE2 is a major PG implicated in inflammatory reactions [11–13] and contributes to adipose tissue metabolism [14]. The IPFP releases higher levels of PGE2 than subcutaneous adipose tissue in vitro [15]. Several studies have reported that mPGES-1 expression and PGE2 production are lower in human adipocytes derived from obese patients and adipose tissue harvested from high fat diet (HFD) mice than those from their non-obese and control counterparts, respectively [16]. Investigation of the arachidonic acid cascade in the IPFP of OA patients with HCE may unveil a possible mechanism for HCE in the initiation and/or development of OA.

Here, we examined the expression levels of various inflammatory meditators in the IPFP of OA patients with HCE.

## Methods

We studied samples from 31 men and 114 women (mean ± standard deviation [SD] age = 73.2 ± 7.7 years; body mass index [BMI] = 26.2 ± 4.2 kg/m^2^) with knee OA diagnosed by radiography (unilateral Kellgren/Lawrence grades 2 [*n* = 3/145, 2%], 3 [*n* = 56/145, 40%], and 4 [*n* = 86/145, 58%]; serum total cholesterol (TCHO): = 207 ± 39 mg/dl; serum triglycerides [TG] = 129 ± 70 mg/dl; and serum hemoglobin 1Ac [HbA1c] = 6.0 ± 0.5%). All participants received total knee arthroplasty at our institution from March 2015 to June 2017. IPFP and synovial tissue (ST) specimens were extracted from each operated knee during the surgery. A piece of each IPFP and ST specimen was instantly frozen in liquid nitrogen at − 80 °C until RNA extraction.

This study was approved by the Ethics Review Board of Kitasato University (reference number: KMEO B13–113). Informed consent was obtained from all participants the day before surgery for participation in this study and the extraction and use of their ST.

### Real-time (RT)-polymerase chain reaction (PCR) analysis

OA patients were divided into three groups according to their TCHO levels (Desirable, Borderline high and High) based on the National Cholesterol Education Program Adult Treatment Panel III (NCEP ATP III) (Table 1). Clinical characteristics of patients in each group are shown in Table 2. Total RNA extraction, cDNA synthesis and real-time PCR methods were conducted as described previously [17]. Primers used are listed in Table 3. We examined the expression of COX-2, mPGES1, TNF-α, IL-1β, and IL-6 in the IPFP and ST using real-time PCR and compared these among the three groups. We also divided the patients into three groups (normal, overweight, obese) based on the World Health Organization Body Mass Index (BMI) classification (Additional files 1 and 2: Tables S1 and S2). However, we observed no differences in COX-2 or mPGES1 expression in the IPFP among the BMI groups (Additional file 3: Figure S1).

**Table 1:** NCEP ATP III classification of total cholesterol

Total cholesterol (mg/dl)	Classification
< 200	Desirable
200–239	Borderline high
≥240	High

**Table 2:** Clinical characteristics of patients classified into three groups according to total cholesterol level

	Desirable (*n* = 60)	Borderline high (*n* = 61)	High (*n* = 24)	*P*
Age (years)	73.5 ± 7.3	72.9 ± 8.6	73.6 ± 6.8	0.912
Male/Female, n	16/44	11/50	4/20	0.453
KL (2/3/4)	1/24/35	1/24/36	1/8/15	0.861
BMI (kg/m^2^)	26.6 ± 4.2	26.2 ± 4.4	25.1 ± 3.5	0.360
TCHO (mg/dl)	173 ± 22	218 ± 12	267 ± 32	< 0.001
TG (mg/dl)	124 ± 83	127 ± 49	145 ± 81	0.436
HbA1c (%)	6.0 ± 0.5	6.0 ± 0.5	5.8 ± 0.3	0.287

All values indicate mean ± standard deviation unless otherwise indicated

*KL* Kellgren and Lawrence grade, *BMI* body mass index, *TCHO* total cholesterol, *TG* triglyceride, *HbA1c* hemoglobin A1c

**Table 3:** Primer sequences

Primer	Sequence (5′–3′)	Product size (bp)
COX-2-F	TGGCTGAGGGAACACAACAG	74
COX-2-R	AACAACTGCTCATCACCCCA	
mPGES1-F	GGAGACCATCTACCCCTTCCT	81
mPGES1-R	AAGTGCATCCAGGCGACAAA	
TNF-α-F	CCCATCCCATCTTCCACAGG	74
TNF-α-R	GGTGGTCTTATCCCCAACCC	
IL-1β-F	GTACCTGTCCTGCGTGTTGA	153
IL-1β-R	GGGAACTGGGCAGACTCAAA	
IL-6-F	GAGGAGACTTGCCTGGTGAAA	199
IL-6-R	TGGCATTTGTGGTTGGGTCA	
GAPDH-F	TGTTGCCATCAATGACCCCTT	202
GAPDH-R	CTCCACGACGTACTCAGCG	

### Statistical analysis

Statistical analysis was performed using the SPSS 19.0 statistical package. Analysis of variance and Tamhane’s T2 test were used for continuous variables, and the Fisher exact test was used for categorical variables. A *p* value of 0.05 was considered statistically significant.

## Results

**Fig. 1:**
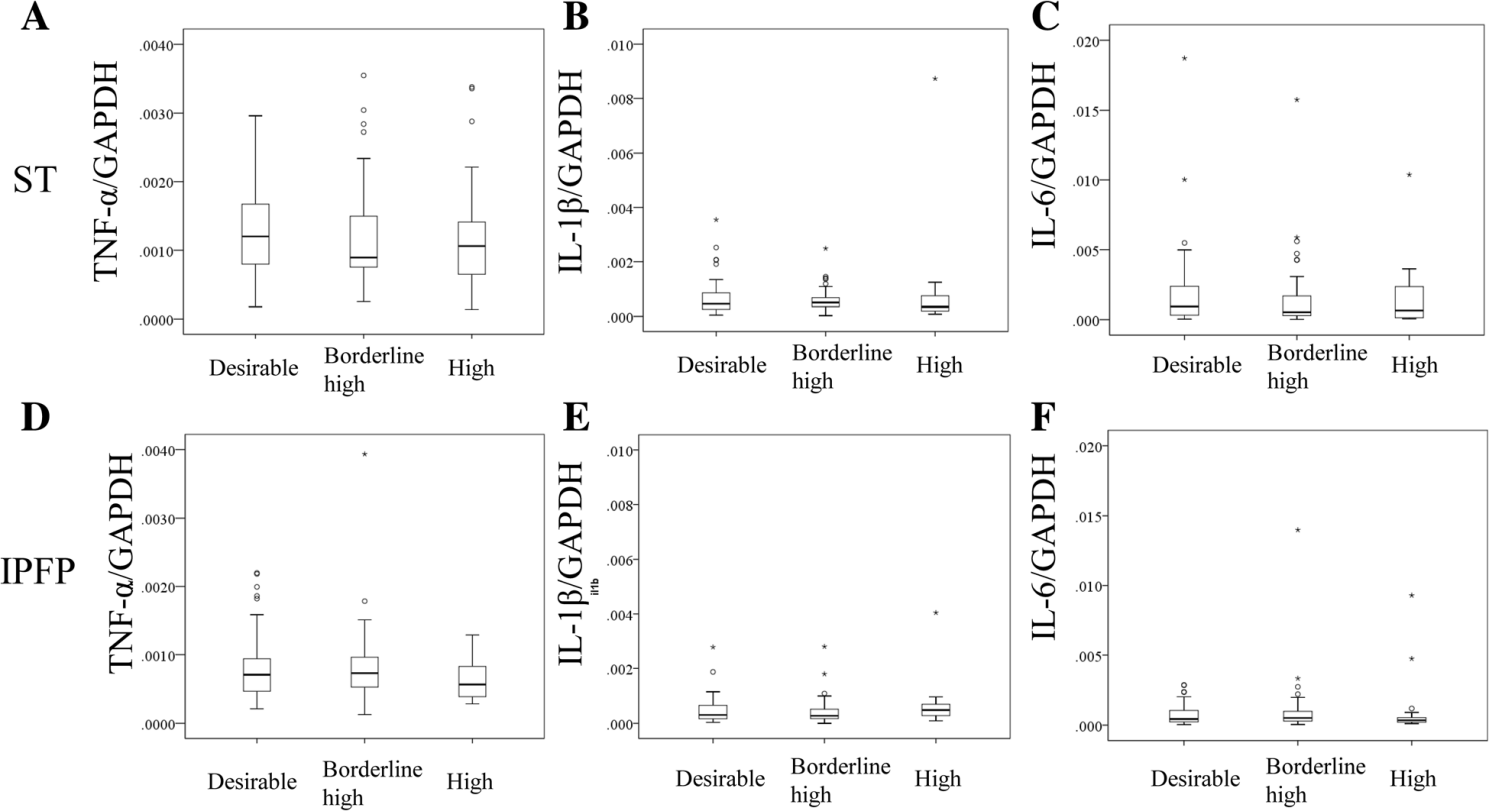
Effect of cholesterol level on inflammatory cytokine expression in synovial tissues (ST) and the infrapatellar fat pad (IPFP). Tumor necrosis factor (TNF)-α expression in ST (**a**) and the IPFP (**d**). Interleukin (IL)-1β expression in ST (**b**) and the IPFP (**e**). Interleukin (IL)-6 expression in ST (**c**) and the IPFP (**f**). GAPDH, glyceraldehyde-3-phosphate dehydrogenase

**Fig. 2:**
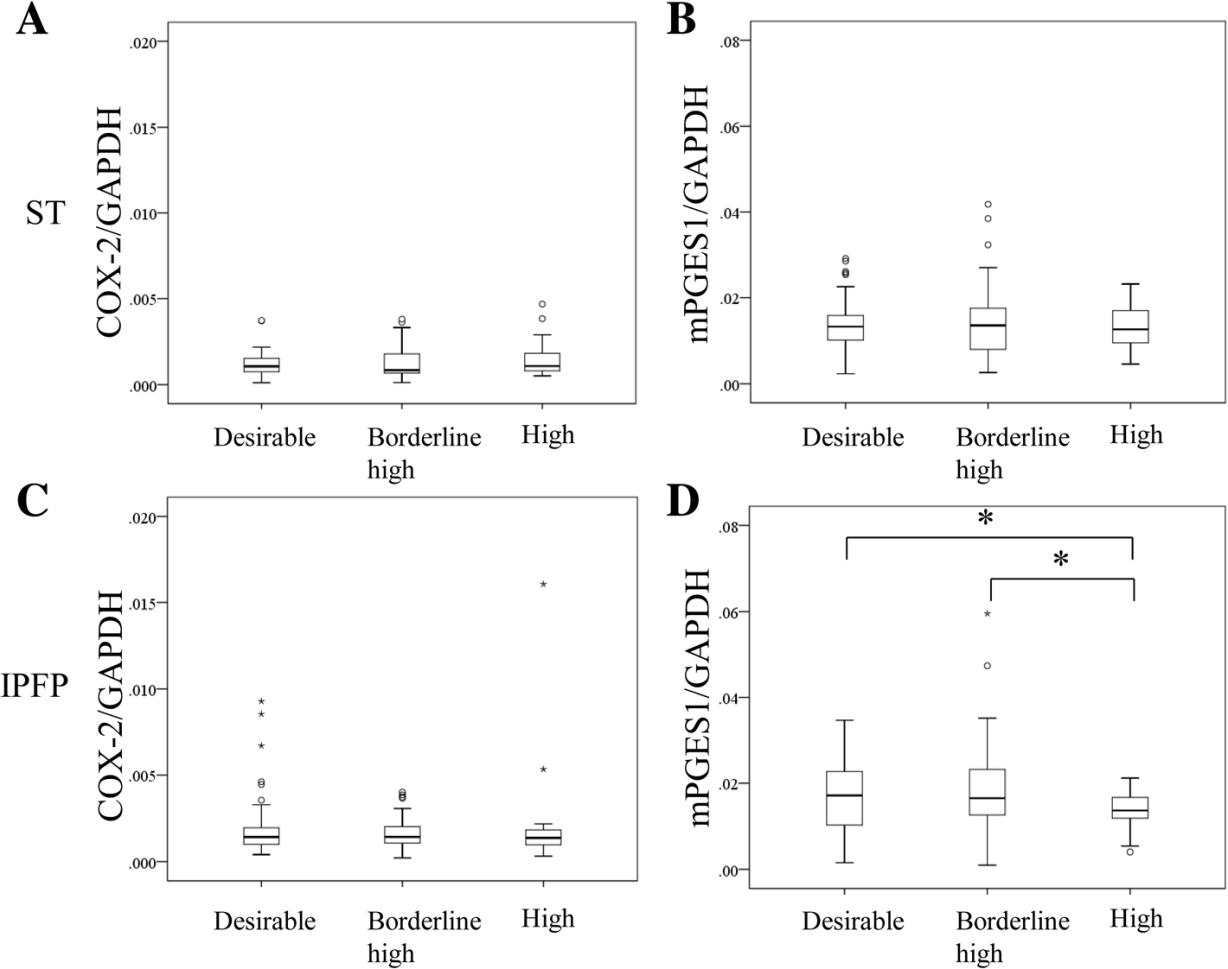
Effect of cholesterol level on cyclooxygenase-2 (COX-2) and microsomal prostaglandin E synthase-1 (mPGES1) expression in synovial tissues (ST) and the infrapatellar fat pad (IPFP). COX-2 expression in ST (**a**) and the IPFP (**c**). mPGES1 expression in ST (**b**) and the IPFP (**d**). GAPDH, glyceraldehyde-3-phosphate dehydrogenase

Patients divided into three groups according to their TCHO based on ATP III showed no differences in age, male/female ratio, KL grade ratio, BMI, TG, or HbA1c (Table 1). The expression levels of TNF-α, IL-1β, and IL-6 in ST and the IPFP were not significantly different among the three groups (Fig. 1a–f). COX-2 mRNA expression in ST and the IPFP was likewise not different among the three groups (Fig. 2a, c). While the mRNA expression level of mPGES1 in ST was also not significantly different (Fig. 2b), that of mPGES1 in the IPFP was significantly lower in the High group than the Desirable and Borderline high groups (Fig. 2d).

## Discussion

Several studies have reported that inflammatory cytokine levels in the IPFP are affected by obesity. Elevated TNF-α expression has been observed in the IPFP of HFD mice [18]. Moreover, TNF-α levels are increased in the fat-conditioned medium (FCM) obtained from the IPFP of patients with high BMI (BMI > 30) compared to that from patients with low BMI (BMI ≤25) [19]. We observed no difference in the mRNA expression of TNF-α, IL-1β, or IL-6 among Desirable, Borderline high and High cholesterol groups. HCE in HFD mice is commonly accompanied by obesity and hyperlipidemia, and HFD models are used to examine the development of obesity. Taken together, our results and those of previous studies suggest that elevated inflammatory cytokine production in the IFPF may be affected by BMI rather than HCE.

Several studies have reported that the arachidonic cascade is altered in several metabolic conditions [14, 16]. Cultured adipocytes from obese rats release PGE at lower rates than those from lean rats [14]. Further, a higher degree of adipocyte differentiation in culture is correlated with reduced basal PGE synthesis for cells derived from obese compared to lean rats [14]. The release of PGE2 by adipocytes from human visceral and subcutaneous abdominal adipose tissues is markedly reduced in excessively obese individuals (BMI > 45 kg/m^2^). The expression of mPGES1 is selectively reduced in WAT of HFD mice [16]. We similarly observed a decrease in the expression of mPGES1 in the IPFP of OA patients with HCE, although no difference was observed in mPGES1 levels among normal, overweight, and obese patients. HCE may therefore affect the production of PGE2 in the IPFP.

A number of studies on articular cartilage or chondrocytes have concluded that the inhibition or stimulation of PGE2 concentration by various molecules or factors indicates chondro-protective or chondro-destructive effects by those molecules or factors, respectively [20]. PGE2 has similarly been implicated in inflammation and joint destruction in animal arthritis models [21, 22]. mPGES-1 expression by inflammatory stimuli correlates with increased PGE2 production [23]. mPGES-1 is located in cartilage in OA patients [24]. Further, mPGES-1 mRNA and protein levels in OA cartilage were increased compared to normal cartilage [24]. Recent studies have reported that inhibition of mPGES1 relieves pain in mice and canine OA models, suggesting that mPGES1 might be a therapeutic target in OA [13, 25, 26]. In contrast, a few reports have shown that continuous inhibition of PGE2 using nonsteroidal anti-inflammatory drugs accelerates OA progression [27, 28]. Moreover, PGE2 reportedly has both anabolic and catabolic functions in chondrocytes [29, 30]. Continuous suppression of mPGES-1 in the IPFP of HCE patients may reduce PGE2 production. Further studies are required to confirm this and to clarify how this affects the knee joint.

There are two main limitations of this study. First, we did not examine the expression levels of inflammatory mediators in a control, non-OA patient population; further studies comparing OA and non-OA populations are needed to confirm our findings. Second, we did not examine the relationship between OA pathology and mPGES1 expression.

## Conclusions

Expression levels of mPGES-1 are reduced in the IPFP of knee OA patients with HCE. Additional studies are needed to clarify the effect of mPGES-1 down-regulation in OA pathology.

## Additional files

Additional file 1: **Table S1.** World Health Organization Body Mass Index (BMI) classification. (DOCX 14 kb)

Additional file 2: **Table S2.** Clinical characteristics of patients classified into three groups according to their body mass index. (DOCX 15 kb)

Additional file 3: **Figure S1.** Effect of body mass index on cyclooxygenase-2 (COX-2) and microsomal prostaglandin E synthase-1 (mPGES1) expression in synovial tissues (ST) and the infrapatellar fat pad (IPFP). OA patients (*n* = 145) were divided into three groups (normal, overweight, obese) according to the WHO BMI classification. We examined the expression of COX-2 and mPGES1 in the IPFP and ST using real-time PCR and compared these among normal, overweight, and obese groups. There were no differences in COX-2 or mPGES1 expression in the IPFP or in ST among the groups. GAPDH, glyceraldehyde-3-phosphate dehydrogenase. (TIFF 1428 kb)

## Abbreviations

BMI: Body mass index
COX-2: Cyclooxygenase-2
FCM: Fat-conditioned medium
GAPDH: Glyceraldehyde-3-phosphate dehydrogenase
HCE: Hypercholesterolemia
HFD: High fat diet
IL-1β: Interleukin-1β
IL-6: Interleukin-6
mPGES-1: Microsomal prostaglandin E synthase-1
NCEP ATP III: National Cholesterol Education Program Adult Treatment Panel III
OA: Osteoarthritis
PCR: Polymerase chain reaction
PGE2: Prostaglandin E2
TNF-α: Tumor necrosis factor-α
