# Supplementary material for: Down-regulation of microsomal prostaglandin E2 synthase-1 in the infrapatellar fat pad of osteoarthritis patients with hypercholesterolemia
Source: Lipids Health Dis. 2018 Jun 13;17:137. doi: 10.1186/s12944-018-0792-7 (PMC6001124; doi:10.1186/s12944-018-0792-7)
Supplement: Supplementary file 1 — Table S1. World Health Organization Body Mass Index (BMI) classification. (DOCX 14 kb) [file 12944_2018_792_MOESM1_ESM.docx]

Table S1 World Health Organization Body Mass Index (BMI) classification

| BMI (kg/m^2^) | Classification |
| --- | --- |
| <25 | Normal |
| 25-29.9 | Overweight |
| ≥30 | Obese |
