# Supplementary material for: Down-regulation of microsomal prostaglandin E2 synthase-1 in the infrapatellar fat pad of osteoarthritis patients with hypercholesterolemia
Source: Lipids Health Dis. 2018 Jun 13;17:137. doi: 10.1186/s12944-018-0792-7 (PMC6001124; doi:10.1186/s12944-018-0792-7)
Supplement: Supplementary file 2 — Table S2. Clinical characteristics of patients classified into three groups according to their body mass index. (DOCX 15 kb) [file 12944_2018_792_MOESM2_ESM.docx]

Table S2 Clinical characteristics of patients classified into three groups according to their body mass index

|  | Normal  (n=60) | Overweight  (n=60) | Obese  (n=25) | P |
| --- | --- | --- | --- | --- |
| Age (years) | 75.3 ± 7.6 | 72.5 ± 7.1 | 69.7 ± 8.4 | 0.007 |
| Male/Female, n | 10/50 | 19/41 | 2/23 | 0.030 |
| KL (2/3/4) | 1/20/39 | 2/27/31 | 0/9/16 | 0.598 |
| BMI (kg/m^2^) | 26.6 ± 4.2 | 26.2 ± 4.4 | 25.1 ± 3.5 | <0.001 |
| TCHO (mg/dl) | 173 ± 22 | 218 ± 12 | 267 ± 32 | 0.080 |
| TG (mg/dl) | 124 ± 83 | 127 ± 49 | 145 ± 81 | 0.051 |
| HbA1c (%) | 6.0 ± 0.5 | 6.0 ± 0.5 | 5.8 ± 0.3 | <0.001 |

KL, Kellgren and Lawrence grade; BMI, body mass index; TCHO, total cholesterol; TG, triglyceride; HbA1c, hemoglobin A1c.

All values indicate mean ± standard deviation unless otherwise indicated
