# Supplementary figures and images for: Down-regulation of microsomal prostaglandin E2 synthase-1 in the infrapatellar fat pad of osteoarthritis patients with hypercholesterolemia
Source: Lipids Health Dis. 2018 Jun 13;17:137. doi: 10.1186/s12944-018-0792-7 (PMC6001124; doi:10.1186/s12944-018-0792-7)

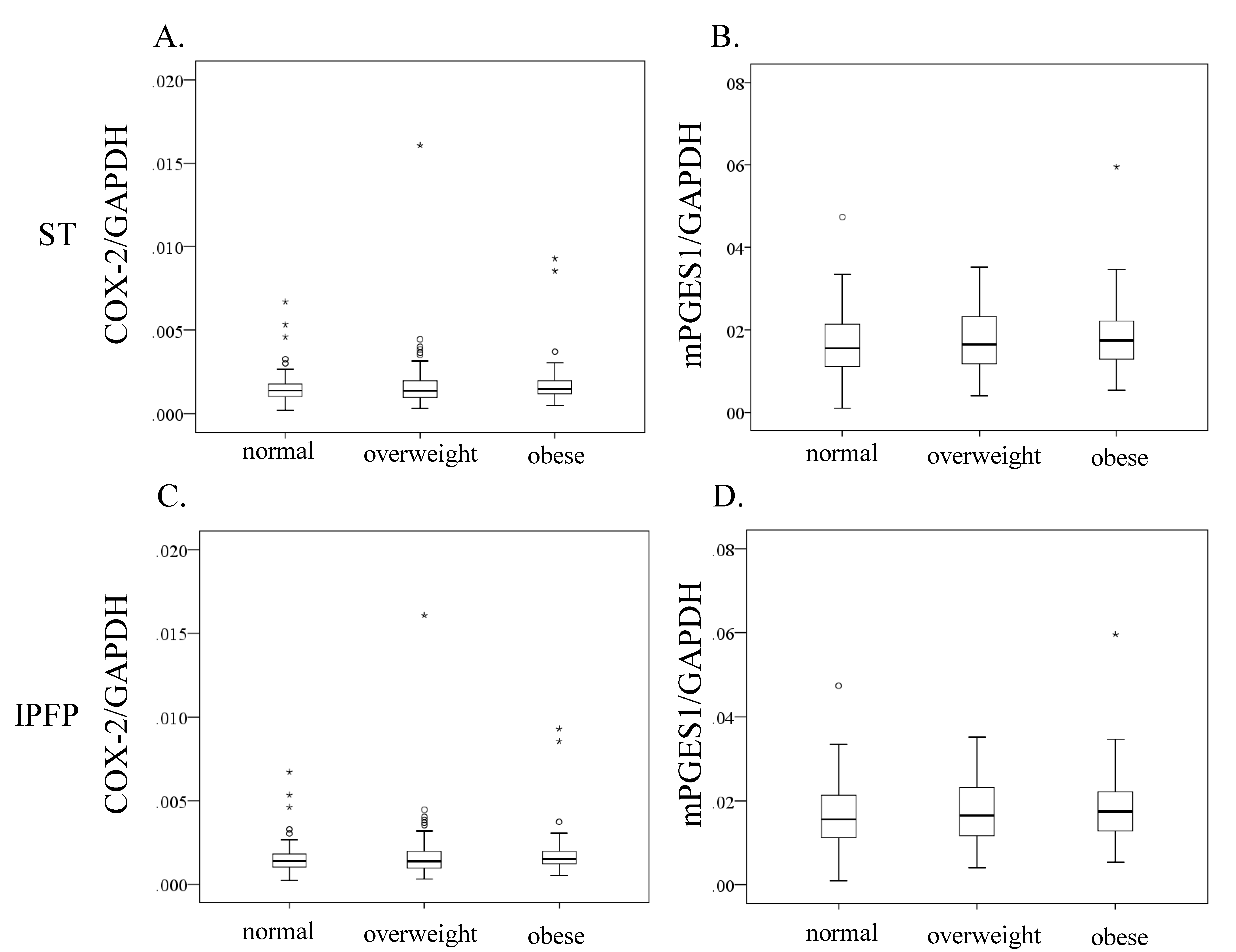

Supplement: Supplementary file 3 — Figure S1. Effect of body mass index on cyclooxygenase-2 (COX-2) and microsomal prostaglandin E synthase-1 (mPGES1) expression in synovial tissues (ST) and the infrapatellar fat pad (IPFP). OA patients (n = 145) were divided into three groups (normal, overweight, obese) according to the WHO BMI classification. We examined the expression of COX-2 and mPGES1 in the IPFP and ST using real-time PCR and compared these among normal, overweight, and obese groups. There were no differences in COX-2 or mPGES1 expression in the IPFP or in ST among the groups. GAPDH, glyceraldehyde-3-phosphate dehydrogenase. (TIFF 1428 kb) [file 12944_2018_792_MOESM3_ESM.tiff]
